# Hispolon Protects against Acute Liver Damage in the Rat by Inhibiting Lipid Peroxidation, Proinflammatory Cytokine, and Oxidative Stress and Downregulating the Expressions of iNOS, COX-2, and MMP-9

**DOI:** 10.1155/2012/480714

**Published:** 2011-10-15

**Authors:** Guan-Jhong Huang, Jeng-Shyan Deng, Chuan-Sung Chiu, Jung-Chun Liao, Wen-Tsong Hsieh, Ming-Jyh Sheu, Chieh-Hsi Wu

**Affiliations:** ^1^School of Chinese Pharmaceutical Sciences and Chinese Medicine Resources, College of Pharmacy, China Medical University, Taichung 404, Taiwan; ^2^Department of Health and Nutrition Biotechnology, Asia University, Taichung 413, Taiwan; ^3^Nursing Department, Hsin Sheng College of Medical Care and Management, Taoyuan 325, Taiwan; ^4^School of Pharmacy, College of Pharmacy, China Medical University, Taichung 404, Taiwan; ^5^Department of Pharmacology, China Medical University, Taichung 404, Taiwan

## Abstract

The hepatoprotective potential of hispolon against carbon tetrachloride (CCl_4_)-induced liver damage was evaluated in preventive models in rats. Male rats were intraperitoneally treated with hispolon or silymarin once daily for 7 consecutive days. One hour after the final hispolon or silymarin treatment, the rats were injected with CCl_4_. Administration with hispolon or silymarin significantly decreased the alanine aminotransferase (ALT) and aspartate aminotransferase (AST) levels in serum and increased the activities of superoxide dismutase (SOD), catalase, glutathione peroxidase (GPx), and glutathione (GSH) content and decreased the malondialdehyde (MDA) content in liver compared with CCl_4_-treated group. Liver histopathology also showed that hispolon reduced the incidence of liver lesions induced by CCl_4_. In addition, hispolon decreased nitric oxide (NO) production and tumor necrosis factor (TNF-**α**), inducible NO synthase (iNOS) and cyclooxygenase-2 (COX-2) activation in CCl_4_-treated rats. We also examined the involvement of matrix metalloproteinase (MMP)-9 in the development of CCl_4_-induced liver damage in rats. Hispolon inhibited the expression of MMP-9 protein, indicating that MMP-9 played an important role in the development of CCl_4_-induced rat liver damage. Therefore, we speculate that hispolon protects rats from liver damage through their prophylactic redox balancing ability and anti-inflammation capacity.

## 1. Introduction

It has been reported that liver injuries induced by carbon tetrachloride (CCl_4_) are the best characterized system of xenobiotic-induced hepatotoxicity and commonly used model for the screening of antihepatotoxic and hepatoprotective activities of drugs [1]. The principal cause of CCl_4_-induced hepatic damage involves increased lipid peroxidation, decreased activities of antioxidant enzymes, and biotransformation of free radical derivatives [2]. CCl_4_-induced hepatotoxicity is believed to involve two phases. The first involves cytochrome P450 2E1-mediated metabolism of toxic metabolite trichloromethyl radical (CCl_3_^∙^) to a highly reactive trichloromethyl peroxy radical (CCl_3_OO^∙^), which initiates lipid peroxidation and leads to peroxidative degradation of cellular membrane leading to the necrosis of hepatocytes [3]. The second step involves the activation of Kupffer cells, probably by free radicals [4]. The activation of Kupffer cells is accompanied by the production of proinflammatory mediators [5]. As a result of the hepatic injury, the altered permeability of the membrane causes the enzymes within the cells to be released into circulation, which damages the hepatic cells, as shown by the abnormally high level of serum hepatospecific enzymes.

Those inflammatory mediators from activated hepatic macrophages are thought to potentiate CCl4-induced hepatic damage [6], and macrophages could be involved in the release of a number of inflammatory mediators contributing to xenobiotic-induced tissue injury [7]. Two mediators of interest are tumor necrosis factor-*α* (TNF-*α*) and nitric oxide (NO). TNF-*α* is unique among cytokines in which it can induce cytotoxicity directly and has been implicated in apoptosis [8]. NO is a highly reactive oxidant, produced by parenchymal and nonparenchymal liver cells from L-arginine via the action of inducible nitric oxide synthase (iNOS) [9]. When released by macrophages against infectious agents, nitric oxide has been shown to inhibit mitochondrial respiration and DNA synthesis [10]. Macrophages and inflammatory mediators including TNF-*α* and NO have been implicated in liver damage induced by a number of different toxicants [7]. 

Matrix metallopeptidases (MMPs), a family of zinc- and calcium-dependent proteinases, participates in the degradation of extracellular matrix (ECM) [11]. Various cell types such as neutrophils, macrophages, and hepatic satellite cells have been demonstrated to produce MMPs around hepatocyte damaged sites [12]. Recently, increasing discoveries of activities involving MMP-2 and -9 in the liver have been reported in animal hepatitis models [13]. Therefore, a broad-spectrum MMP inhibitor may be potentially useful for the treatment of patients with acute and perhaps chronic liver failure.

*Phellinus linteus* (Berk. & M.A. Curt.) (PL) is a mushroom that belongs to the genus *Phellinus* and is commonly called “Sangwhang” in Taiwan. It is popular in oriental countries and has been traditionally used as food and medicine. PL contains many bioactive compounds and is known to improve health and to prevent and to remedy various diseases, such as gastroenteric disorders, lymphatic diseases, and cancer [14]. We recently reported that hispolon, a phenol compound isolated from PL, anti-inflammatory, antiproliferative, and antimetastatic effects [15]. The objective of this study was to understand more about the protective effects of hispolon on CCl4-induced rat liver damage. In addition, we examined the enzyme expression of MMP-9 in CCl4-induced rat liver damage and the effect of hispolon on MMP-9 expression in this model.

## 2. Materials and Methods

### 2.1. Chemicals

CCl_4_ was purchased from Merck (Germany). Silymarin and other chemicals were purchased from Sigma Chemical Co (Steinheim, Germany). Biochemical assay kits (ALT kit; AST kit; SOD kit; GPx kit) were purchased from Randox Laboratories (Crumlin, UK). TNF-*α* was purchased from Biosource International Inc., (Camarillo, Calif, USA). The antibody against iNOS, COX-2, MMP-9, and *β*-actin were purchased from Cell Signaling Technology (Beverly, Mass, USA). 

### 2.2. Isolation and Characterization of Hispolon from Fruiting Body of PL

The fruiting body of PL (about 1.0 kg, air dry weight) was powdered and extracted with 95% EtOH 6 L at room temperature (3 times, 72 h each). Extracts were filtered and combined together and then evaporated at 40°C (N-11, Eyela, Japan) to dryness under reduced pressure to give a dark brown residue (40 g). The yield obtained for PL is about 4%. The crude extract was suspended in H_2_O (1 L), and then partitioned with 1 L *n*-hexane (×2), 1 L EtOAc (×2), and 1 L *n*-butanol (×2), successively. 

**Figure 1:**
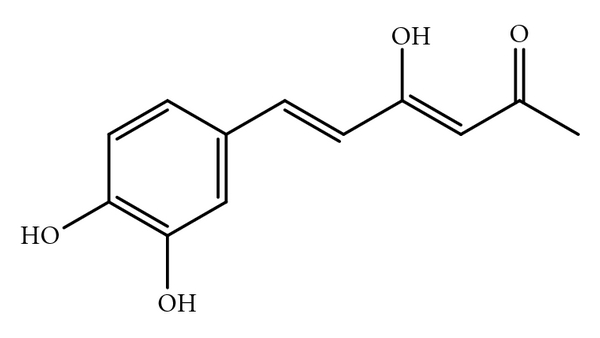
Chemical structure of hispolon.

Hispolon was purified from the EtOAc soluble portion (8 g) by a bioassay-guid separation. A portion of the active EtOAc fraction was subjected to silica gel chromatography using stepwise CHCl_3_-MeOH (9 : 1, 8 : 2, 1 : 1 v/v) as eluent. Final purification was achieved by preparative HPLC (Spherisorb ODS-2 RP18, 5 *μ*m; Promochem), 250 × 25 mm, acetonitrie-H_2_O (83 : 17 v/v), at a flow rate of 10 mL/min and UV detection at 375 nm. The identification of hispolon (Figure 1) was performed by comparing their physical spectral data with the literature values [16].

### 2.3. Animal Treatment

Male Sprague-Dawley (SD) rats, weighing 180–200 g (6–8 weeks old), were maintained at 25 ± 2°C with a 12 h light/dark cycle and fed with a standard rat chow and water *ad libitum*. Being divided into six groups (*n* = 8), normal and negative control groups were orally administered with distilled water. The positive control group was orally administered with silymarin (200 mg/kg in 1% carboxymethyl cellulose) once daily for 7 days. In the three experimental groups, the rats were intraperilotneally (*i.p.*) pretreated with hispolon (5, 10, and 20 mg/kg, *i.p.*) once daily for seven consecutive days. One hour after the last treatment of hispolon and silymarin, all the rats were treated with CCl_4_ (1.5 mL/kg in olive oil, 20%, *i.p.*). 24 h after the CCl_4_ treatment, animals were anesthetized with ethyl ether and blood samples were collected through their carotid arteries. Animal studies were conducted according to the regulations of the Institutional Animal Ethics Committee (IAEC) of China Medical University, Taichung, Taiwan.

### 2.4. Assessment of Liver Functions

To assess hepatotoxicity, serum activity of AST and ALT were measured using spectrophotometric diagnostic kits according to the manufacturer's recommendations. The blood was centrifuged at 1700 × g (Beckman GS-6R, Germany) at 4°C for 30 minutes to separate serum. ALT and AST were analyzed. Liver tissues collected from the animals were stored at 10% formalin for histopathological studies. Also, liver tissues were kept under −80°C for further analysis of their enzyme levels. The biochemical parameters were analyzed by Spectrophotometer (Roche Cobas Mira plus, Germany) using clinical test kits.

### 2.5. Histopathological Examination

Small pieces of liver, fixed in 10% buffered formalin were processed for embedment in paraffin. Sections of 4-5 *μ*m were cut and stained with hematoxylin and eosin and then examined for histopathological changes under the microscope (Nikon, ECLIPSE, TS100, Japan). Images were taken with a digital camera (NIS-Elements D 2.30, SP4, Build 387) at original magnification of ×200.

### 2.6. Antioxidant Enzyme Activity Measurements

The following biochemical parameters were analyzed to check the hepatoprotective activity of hispolon by our previous methods [17]. Liver homogenates were prepared in cold Tris-HCl (5 mM containing 2 mM EDTA, pH 7.4) using a homogenizer. The unbroken cells and cell debris were removed by centrifugation at 12,000 g for 30 min at 4°C. The supernatant was used immediately for the assays of SOD, catalase, GPx, and GSH. All of these enzymes were determined following the instructions on the Randox Laboratories Ltd kit.

### 2.7. Hepatic Glutathione (GSH) Activity

Hepatic GSH level was determined as described previously [17] with slight modifications. Briefly, 720 *μ*L of the liver homogenate in 200 mM Tris-HCl buffer (pH 7.2) was diluted to 1440 *μ*L with the same buffer. Five percent TCA (160 *μ*L) was added to it and mixed thoroughly. The samples were then centrifuged at 10,000 g for 5 min at 4°C. Supernatant (330 *μ*L) was taken in a tube and 660 *μ*L of Ellman's reagent (DTNB) solution was added to it. Finally the absorbance was taken at 405 nm.

### 2.8. Lipid Peroxidation Intermediates

The malondialdehyde (MDA) content, a measure of lipid peroxidation, was assayed in the form of thiobarbituric acid-reactive substances (TBARS) as previously described [17]. Briefly, 1 g of liver was homogenized in 10 mL of KCl 1.15% (w/v), and the homogenate was filtered through fourfolded gauze. A volume of 0.5 mL of liver homogenate was mixed with 3 mL of H_3_PO_4_ 1% (v/v) and 1 mL of thiobarbituric acid (TBA) 0.6% (w/v) and then heated to and maintained at 100°C for 45 min. The samples were allowed to reach room temperature and 3 mL of *n*-butanol was added. After shaking vigorously with the vortex, the butanolic phase was obtained by centrifugation at 4,000 × g for 10 min to determinate the absorbance at 535 nm. The standard was 1, 1, 1, 3-tetraethoxypropane. The protein concentration of the sample was determined by the Bradford dye-binding assay (Bio-Rad, Hercules, Calif, USA).

### 2.9. Measurement of Serum TNF-*α* by ELISA

Serum levels of TNF-*α* were quantified by using a commercially available enzyme-linked immunosorbent assay (ELISA) kit (Biosource International Inc., Camarillo, Calif, USA.) according to the manufacturer's instruction. TNF-*α* was quantified from a standard curve. The concentrations were expressed as pg/mL.

### 2.10. Measurement of Nitric Oxide/Nitrite

The production of NO was assessed indirectly by measuring the nitrite levels in the plasma by a calorimetric method based on the Griess reaction [17]. Plasma samples were diluted four times with distilled water and deproteinized by adding 1/20 volume of zinc sulfate (300 g/L) to a final concentration of 15 g/L. After centrifugation at 10,000 × g for 5 min at room temperature, 100 *μ*L supernatant was applied to a microtiter plate well, followed by 100 *μ*L of Griess reagent (1% sulfanilamide and 0.1% *N*-1-naphthylethylenediamine dihydrochloride in 2.5% polyphosphoric acid). After 10 min of color development at room temperature, the absorbance was measured at 540 nm with a MicroReader (Hyperion, Inc., FL, U.S.A.). Sodium nitrite (0.5–20 *μ*M) was used to create a standard calibration curve.

### 2.11. Examination Activity of Matrix Metalloproteinase-9 (MMP-9) by Gelatin Zymography

The activity of MMP-9 in the medium was measured by gelatin zymography protease assay as previously described [18]. Briefly, the supernatant of liver homogenization (10 *μ*g) was prepared with SDS sample buffer without boiling or reduction, and then it was subjected to 0.1% gelatin-8% SDS-PAGE electrophoresis. After electrophoresis, the gel was washed with 2.5% Triton X-100 and then incubated in a reaction buffer (40 mM Tris-HCl, pH 8.0; 10 mM CaCl_2_ and 0.01% NaN_3_) at 37°C for 12 h, then the gel was stained with Coomassie brilliant blue R-250.

### 2.12. Protein Lysate Preparation and Western Blot Analysis of iNOS, COX-2, and MMP-9

Liver tissues were homogenized in lysis buffer (0.6% NP-40, 150 mM NaCl, 10 mM HEPES (pH 7.9), 1 mM EDTA, and 0.5 mM PMSF) at 4°C. BSA (bovine serum albumin) was applied as a protein standard. Protein samples (30 *μ*g) were resolved by denaturing sodium dodecyl sulfate-polyacrylamide gel electrophoresis (SDS-PAGE) using standard methods and then were transferred to PVDF membranes by electroblotting and blocking with 1% BSA. The membranes were probed with the primary antibodies, (iNOS, COX-2, MMP-9, and *β*-actin) at 4°C overnight, washed three times with PBST, and incubated for 1 h at 37°C with horseradish peroxidase-conjugated secondary antibodies. The membranes were washed three times and the immunoreactive proteins were detected by enhanced chemiluminescence (ECL) using hyperfilm and ECL reagent (Amersham International PLC, Buckinghamshire, UK). The results of Western blot analysis were quantified by measuring the relative intensity compared to the control using Kodak Molecular Imaging software and represented in the relative intensities.

### 2.13. Statistical Analysis

All data were presented as mean ± S.D. from three independent experiments. Means of triplicates were calculated. Student's *t-*test was used for comparison between two treatments. A difference was considered to be statistically significant when *P* < 0.05, *P* < 0.01, or *P* < 0.001.

## 3. Results

### 3.1. Effects of Hispolon on Serum AST and ALT Activities

**Figure 2:**
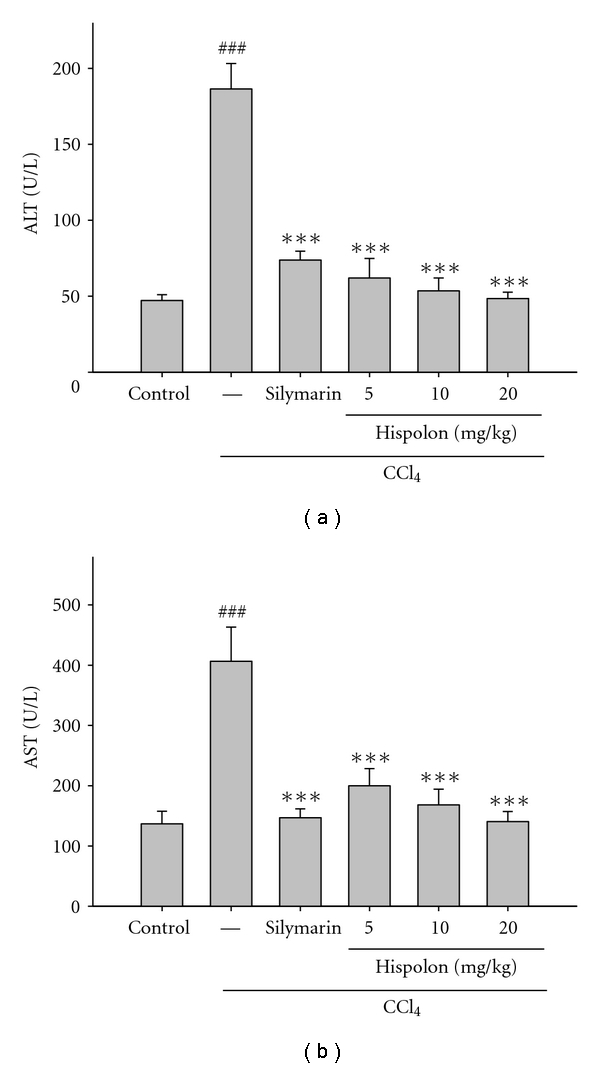
Effect of hispolon on the activities of serum AST and ALT in CCl_4_-injected rats. Animals were *i.p.* treated with hispolon (5, 10, and 20 mg/kg) once daily for consecutive seven days. One hour after the final hispolon treatment, the rats were injected with CCl_4_ (1.5 mL/kg, *i.p.*). The rats were killed 24 h after the CCl_4_ injection. Hepatotoxicity was determined by quantifying the serum activities of aspartate aminotransferase (AST) and alanine aminotransferase (ALT). The values are reported as the mean ± S.D. of six rats per group. ^###^ *P* < 0.001 compared with control; ***P < 0.001 compared with the CCl_4_ group.

Several hepatic enzymes in serum such as AST and ALT in liver homogenate were used as the biochemical markers for the early acute hepatic damage. The levels of AST and ALT were measured in the serum to evaluate hepatic tissue damage. CCl_4_ administration resulted in significant (*P* < 0.001) rise in the levels of AST and ALT when compared with the control group. Preadministration with hispolon (*i.p.*) at three different doses (5, 10, and 20 mg/kg) significantly prevented the increased serum AST and ALT levels. Silymarin (a positive control) at a dose of 200 mg/kg also prevented the elevation of AST and ALT (Figure 2).

### 3.2. Histopathological Analysis

**Figure 3:**
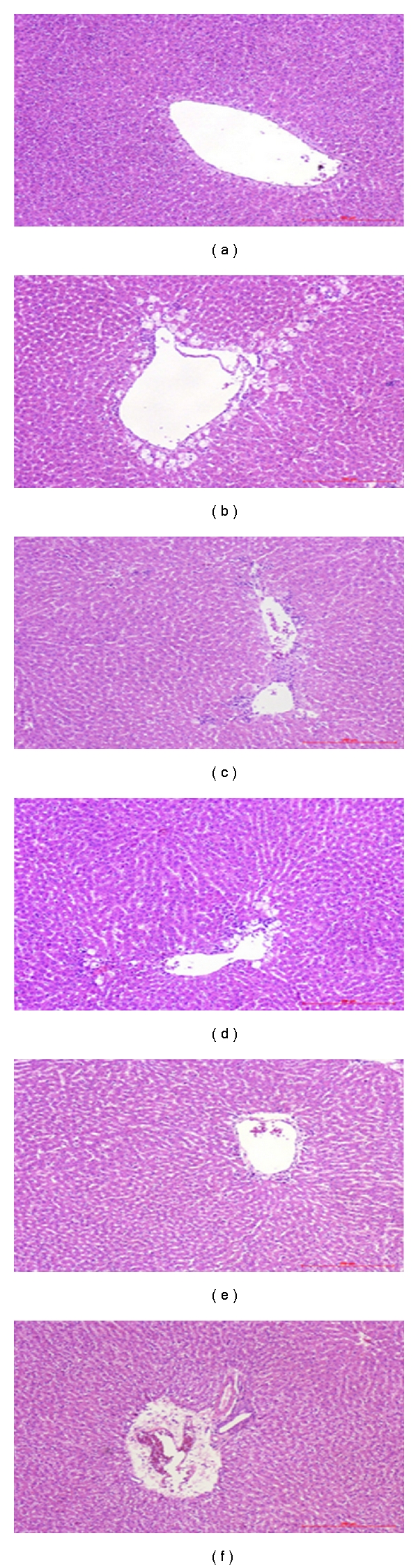
Effect of hispolon on the CCl_4_-induced liver damage. Animals were *i.p.* treated with the hispolon (5, 10, and 20 mg/kg) once daily for consecutive seven days. One hour after the final hispolon treatment, the rats were treated with CCl_4_ (1.5 mL/kg, *i.p.*). Rats were sacrificed 24 h after the CCl_4_ administration and liver was removed, fixed, and embedded in paraffin. Sections were stained with hematoxylin-eosin (×100) and observed under the light microscopy. (a) Control; (b) CCl_4_ (0.5 mL/kg); (c) silymarin (200 mg/kg) + CCl_4_ (0.5 mL/kg); (d) hispolon (5 mg/kg) + CCl_4_ (0.5 mL/kg); (e) hispolon (10 mg/kg) + CCl_4_ (0.5 mL/kg); (f) hispolon (20 mg/kg) +CCl_4_ (0.5 mL/kg).

Results from the histological studies were in agreement on the serum AST and ALT levels. There were no abnormalities or histological changes in the livers of normal rats (Figure 3(a)). Data showed that CCl_4_ induces histological changes including increased degeneration, necrosis, hepatitis, and portal triaditis. Vacuole generation and microvascular steatosis were also observed (Figure 3(b)). Compared with the lesions observed in the CCl_4_ control group, the lesions of silymarin-treated rats were of a much milder degree (Figure 3(c)). Pretreatment with hispolon at 5, 10, and 20 mg/kg reduced the severity of CCl_4_-induced liver intoxication (Figures 3(d), 3(e), and 3(f)). All rats except those in the control group exhibited the ballooning degeneration in the centrolobular zone and the necrosis of hepatocytes (Figure 3). The CCl_4_-induced damage suffered more severely than other groups pretreated with hispolon.

### 3.3. Effect of Hispolon on Hepatic Antioxidant Enzymes Activities

**Figure 4:**
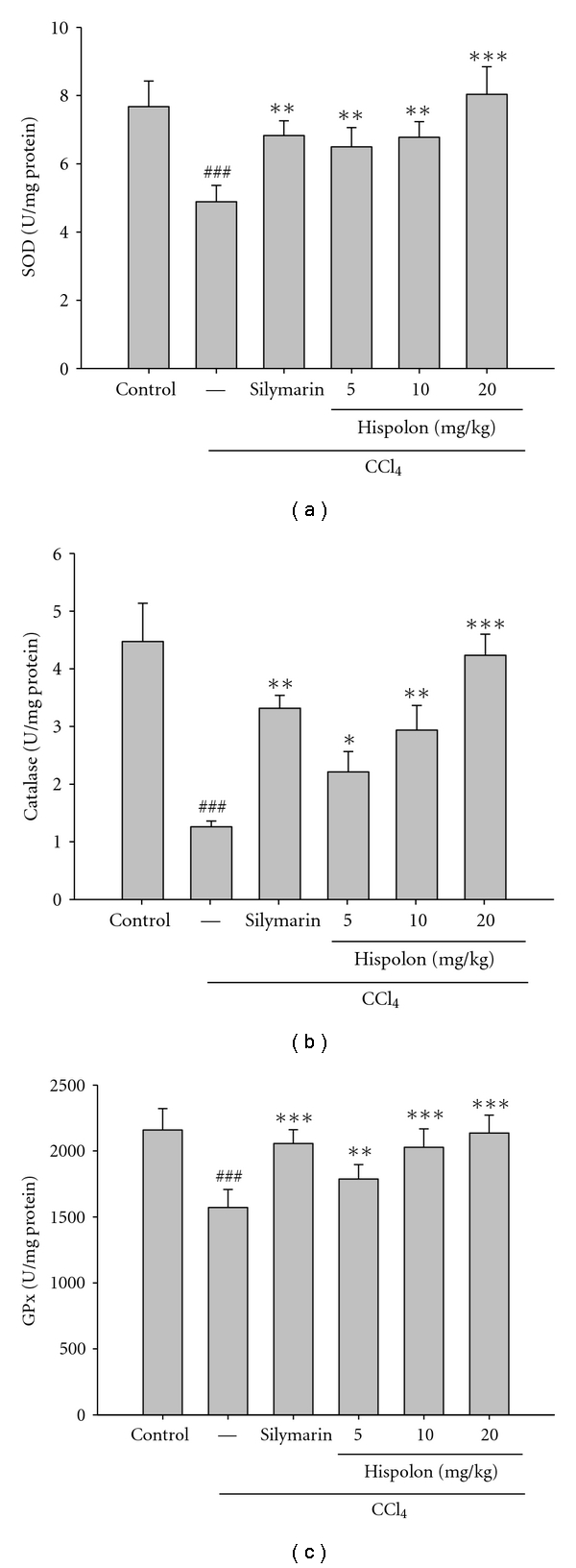
Effect of hispolon on the hepatic antioxidant enzymes (SOD, CAT, and GPx) activities in CCl_4_-treated rats. Animals were *i.p.* treated with hispolon (5, 10, and 20 mg/kg) once daily for consecutive seven days. One hour after the final hispolon treatment, the rats were injected with CCl_4_ (1.5 mL/kg, *i.p.*). The rats were killed 24 h after the CCl_4_ injection. Hepatotoxicity was determined by quantifying the hepatic antioxidant enzymes (SOD, CAT, and GPx). The values are reported as the mean ± S.D. of six rats per group. ^###^ *P* < 0.001, compared with the control group; *P < 0.05, **P < 0.01, and **P < 0.001 compared with the CCl_4_ group.

The superoxide dismutase (SOD), catalase (CAT), and glutathione peroxidase (GPx) activities in CCl_4_-treated rats were significantly decreased, compared to the control. Compared to CCl_4_ group, rats pretreated with hispolon at 5, 10, and 20 mg/kg showed significant increase in SOD (Figure 4(a)), CAT (Figure 4(b)), and GPx (Figure 4(c)), respectively. The reference protective drug silymarin-treated rats also showed significant increase in SOD, CAT, and GPx when it was compared to CCl_4_-treated rats. 

### 3.4. Effect of Hispolon on Lipid Peroxidation in CCl_4_-Treated Rat Liver

**Figure 5:**
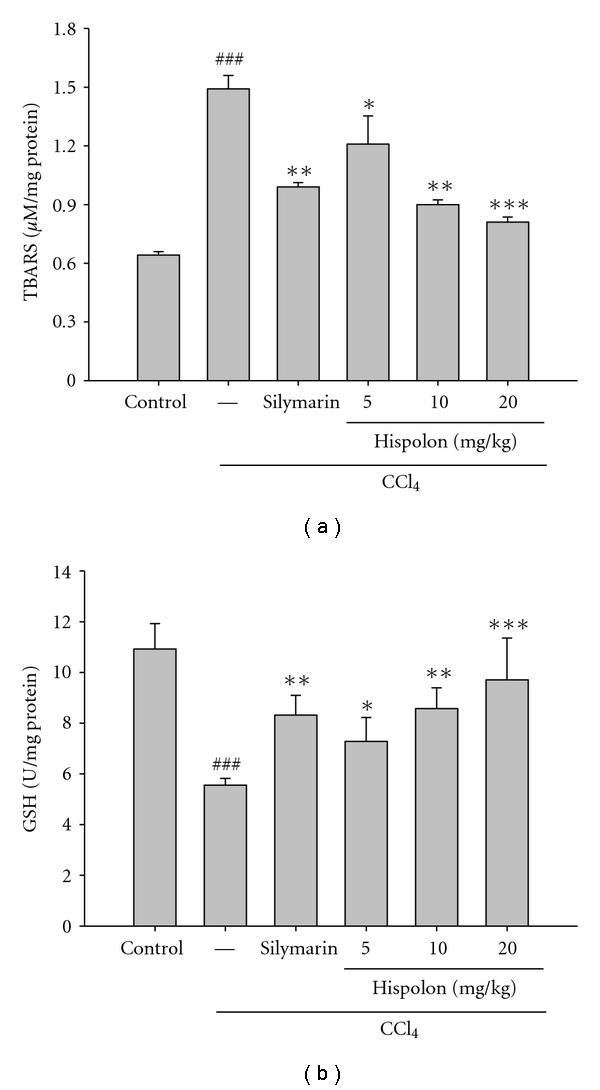
Effect of hispolon on TBARS formation (a) and GSH levels (b) in CCl_4_-injected rats. The values are reported as the mean ± S.D. of six rats per group. ^###^ *P* < 0.001, compared with the control group; *P < 0.05, **P < 0.01, and ***P < 0.001 compared with the CCl_4_ group.

As shown in Figure 5(a), hepatic levels of TBARS were assessed as an indicator of tissue lipid peroxidation. CCl_4_ treatment significantly increased the level of TBARS in the liver. A significant increase (*P* < 0.001) in tissue TBARS level was observed in CCl_4_ alone treated rats. CCl_4_-induced elevation of tissue TBARS concentration was lowered significantly by the *i.p.* pretreatment of the rats with hispolon. Hispolon at 5, 10, and 20 mg/kg significantly prevented the increase in TBARS level when compared to CCl_4_ group. Silymarin also protected the liver from elevating TBARS levels and kept TBARS levels in normal values.

### 3.5. Effects of Hispolon on Hepatic GSH Levels

Because the oxidative stress pathway generally involves the GSH system, we measured the level of GSH in each group. The administration with CCl_4_ significantly depleted GSH (*P* < 0.001), whereas treatment with hispolon (5, 10, and 20 mg/kg) significantly and dose-dependently protected the liver against this effect (Figure 5(b)). Silymarin-treated rats also showed significant increased GSH level in liver homogenates when compared with CCl_4_ group.

### 3.6. Effect of Hispolon on the Serum TNF-*α* Level in CCl_4_-Treated Rats

**Figure 6:**
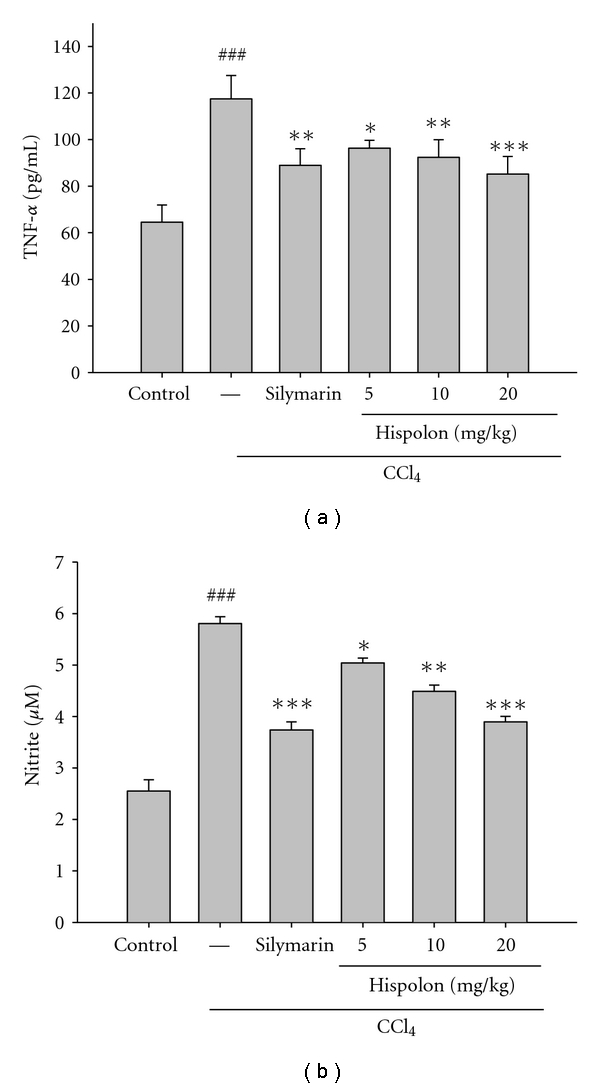
Effect of hispolon on the serum TNF-*α* (a) and NO (b) level in CCl_4_-injected rats. The values are reported as the mean ± S.D. of six rats per group. ^###^ *P* < 0.01 as compared to control group; *P < 0.05, **P < 0.01, and ***P < 0.001 as compared to CCl_4_-treated group.

As shown in Figure 6(a), the level of serum TNF-*α* was 64.59 ± 7.32 pg/mL in control group. CCl_4_ treatment caused significant (*P* < 0.001) increase in the serum TNF-*α* levels when compared with control group. Pretreatment with hispolon at the doses of 5, 10, and 20 mg/kg resulted in significant decrease of TNF-*α* content when compared to CCl_4_-treated rats. Silymarin-treated rats also showed significant (*P* < 0.01) decrease in TNF-*α* level in serum compared with CCl_4_-treated rats.

### 3.7. Effect of Hispolon on Serum NO Production in CCl_4_-Treated Rats

Production of NO in rat serum was significantly enhanced in CCl_4_-treated rats comparing to the control group. However, pretreatment with hispolon concentration dependently decreased the NO production in CCl_4_-treated rats. For example, NO production in the control group was 2.56 ± 0.22 *μ*M/mg protein, while it was 5.81 ± 0.13 *μ*M/mg protein with CCl_4_ treatment. However, the NO production in the CCl_4_-treated rats was significantly (*P* < 0.01) decreased to 5.04 ± 0.09, 4.49 ± 0.12, and 3.89 ± 0.11 *μ*M/mg protein with 5, 10, and 20 mg/kg hispolon oral pretreatment, respectively (Figure 6(b)). Silymarin-(200 mg/kg) treated rats also showed significant (*P* < 0.001) decrease in NO production in serum compared with CCl_4_ group.

### 3.8. Analysis of iNOS/COX-2 Expression Following Hispolon Treatment in Rats with CCl_4_-Induced Liver Injury

**Figure 7:**
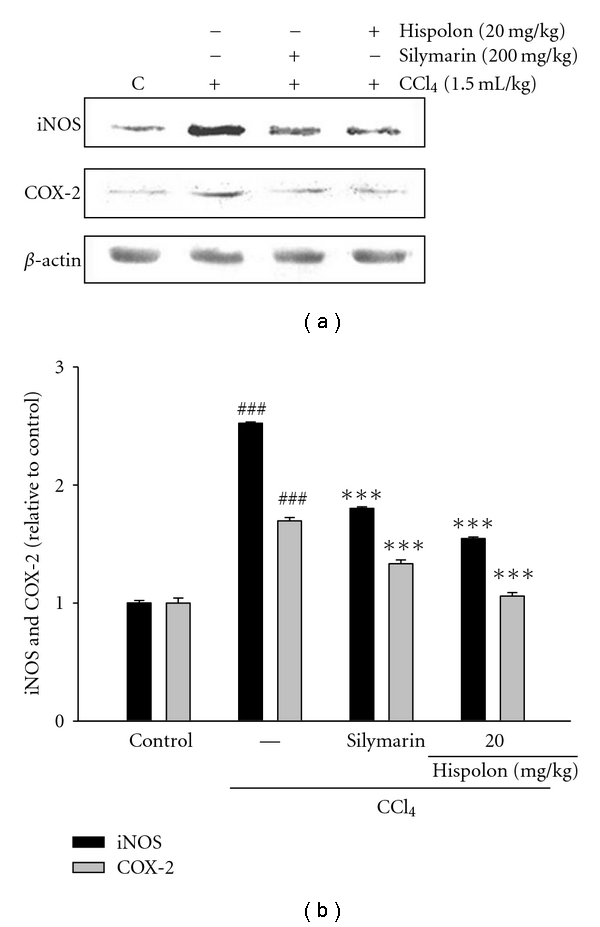
Effect of hispolon on CCl_4_-injected the activation of iNOS (a) and COX-2 (b) in rats. The relative intensities were evaluated by iNOS and COX-2 (c) proteins with the use of the Kodak Molecular Imaging software. *β*-Actin was used as internal control for equal loading of proteins. The values are reported as the mean ± S.D. of six rats per group. ^###^ *P* < 0.001 as compared to control group; ***P < 0.001 as compared to CCl_4_ group.

We investigated the changes of the activation of iNOS and COX-2 by hispolon in CCl_4_-treated rats (Figure 7). The results showed that CCl_4_ treatment stimulates to increase activation of iNOS and COX-2. For example, in CCl_4_ treatment group, the relative intensity of iNOS and COX-2 band was increased by 2.52- and 1.69-fold, compared to the control. However, the treatment of hispolon decreased the iNOS and COX-2 expression in CCl_4_-induced rats. Namely, the relative intensities of bands about iNOS and COX-2 expressions were reduced by 1.55- and 1.06-fold at 20 mg/kg of hispolon, respectively, compared to CCl_4_ treatment alone.

### 3.9. Effect of Hispolon on Serum MMP-9 Activations in CCl_4_-Treated Rats

**Figure 8:**
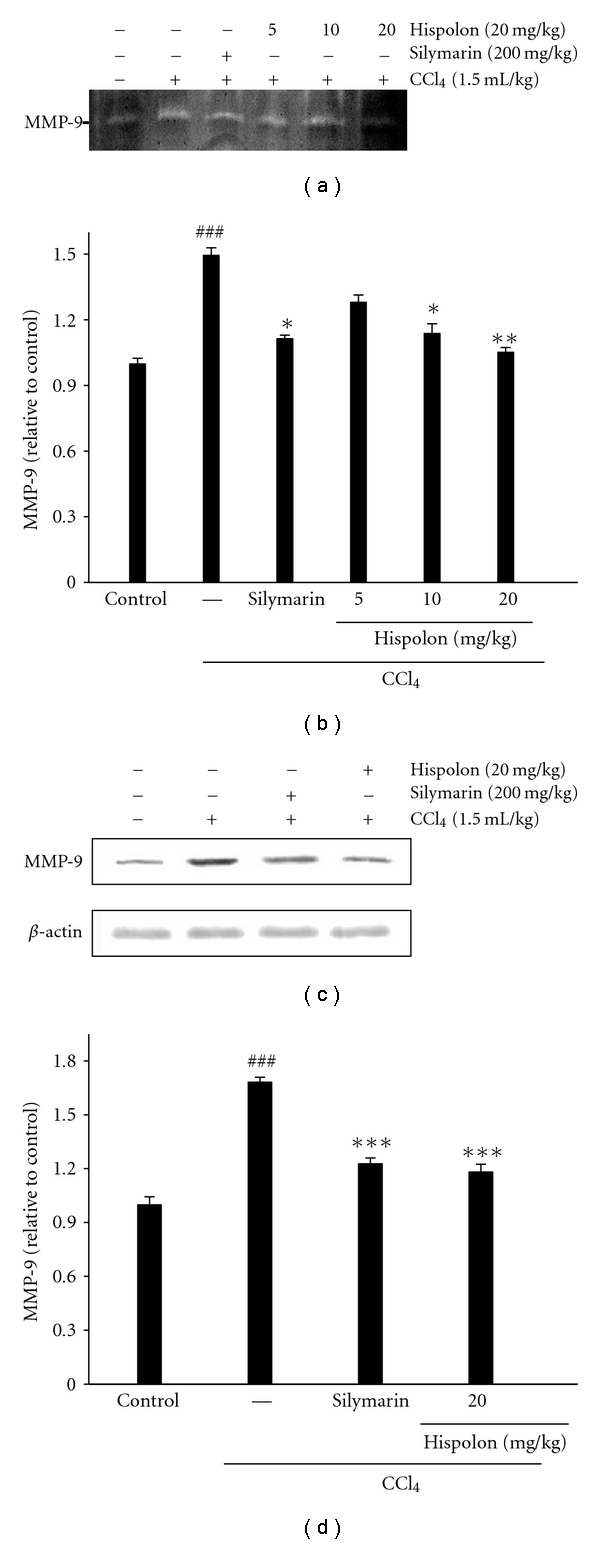
Effect of hispolon on CCl_4_-injected rats the activity of MMP-9 expression. CCl_4_- injected rats were treated with 5, 10, and 20 mg/kg of hispolon for 24 h and then subjected to gelatin zymography to analyze the activities of MMP-9 in the liver (a). The relative intensities were evaluated by MMP-9 (b) proteins with the use of the Kodak Molecular Imaging software. Western blot analysis of hispolon on CCl_4_-injected rats the activation of MMP-9 in rats liver (c). The relative intensities were evaluated by MMP-9 (d) proteins with the use of the Kodak Molecular Imaging software. *β*-actin was used as internal control for equal loading of proteins. The values are reported as the mean ± S.D. of six rats per group.^ ###^ *P* < 0.01 as compared to control group; *P < 0.05, **P < 0.01, and ***P < 0.001 as compared to CCl_4_-treated group.

The effect of hispolon on MMP-9 activation was analyzed by gelatin zymography. As shown in Figure 8(a), MMP-9 secretion was significantly induced by CCl_4_ treatment and suppressed by hispolon (20 mg/kg) pretreatment (*P* < 0.01). In the zymography assay, it was found that hispolon effectively reduced MMP-9 expression in the liver of animals with CCl_4_-induced hepatic damage in a dose-dependent manner (Figure 8(b)).

### 3.10. Effect of MMP-9 Expressions by Hispolon in CCl_4_-Treated Rats Liver

The effect of MMP-9 expression by hispolon in rat liver was assessed by Western blotting. Rats were pretreated with 5, 10, and 20 mg/kg hispolon for 7 days, and induced by CCl_4_ treatment for 24 h. MMP-9 expression was markedly induced in CCl_4_-treated group. And MMP-9 expression was suppressed by hispolon (20 mg/kg) pretreatment (*P* < 0.001) (Figures 8(c) and 8(d)). 

## 4. Discussion

This paper demonstrated that hispolon significantly attenuated CCl_4_-induced hepatotoxicity of the rats. CCl_4_-induced liver damage involved the biotransformation of CCl_4_ by the cytochrome P450 (CYPs) system into a trichloromethyl free radical (CCL_3_^∙^) which causes lipid peroxidation, disrupts Ca_2+_ homeostasis, and eventually kills cells [19]. Many studies have shown that hepatoprotective effects may be associated with an antioxidant capacity [20]. Our results demonstrate that CCl_4_ treatment alone and post-treatment after 24 hours caused severe acute liver damage in rats, as evidenced by increased serum ALT and AST activities (Figure 2). Elevated levels of serums ALT and AST are indication of cellular leakage and loss of functional integrity of cell membrane in liver [20]. The leakage of large quantities of enzymes into the blood stream was associated with massive centrilobular necrosis, ballooning degeneration, and cellular infiltration of the liver. Administration with hispolon decreased the serum levels of ALT and AST towards their respective normal value that is an indication of stabilization of plasma membrane as well as repair of hepatic tissue damage caused by CCl_4_ (Figures 2 and 3). These results imply that hispolon tends to prevent damage and suppressed the leakage of enzymes through cellular membranes, and the phenomenon was confirmed by histological evidences.

Our results demonstrate that hispolon could exert its protective effect against CCl_4_-induced hepatotoxicity. And this could be explained that hispolon enhance antioxidative defense. Our previous studies demonstrate that hispolon decreased the paw edema at the fourth and fifth hours after *λ*-carrageenin administration and increased the activities of SOD, GPx, and glutathione reductase (GRx) in the liver tissue. These anti-inflammatory mechanisms of hispolon might be related to the decrease in the level of MDA in the edema paw by increasing the activities of SOD, GPx, and GRx in the liver. It probably exerts anti-inflammatory effects through the suppression of TNF-*α* and NO [17]. And we also found that hispolon, a phenolic compound from *Phellinus merrillii*, exhibited antioxidant activity. Phenolic compounds possess antioxidant, radical scavenging, and antimutagenic and anticarcinogenic properties [21].

Many studies have demonstrated that antioxidant enzymes such as SOD, CAT, and GPx represent one protection against oxidative tissue damage [21]. SOD is an effective defense enzyme that converts the dismutation of superoxide anions into hydrogen peroxide (H_2_O_2_) [22]. Then, CAT metabolizes H_2_O_2_ to O_2_ and H_2_O. GPx detoxificates the xenobiotics in the liver and catalyses the reduction of H_2_O_2_ and hydroperoxides to nontoxic products. These antioxidant enzymes are inactivated by lipid peroxides or ROS, which results in decreased activities of these enzymes in CCl_4_ toxicity [23]. In the present study, SOD, CAT, and GPx activities were significantly decreased in the liver in response to CCl_4_ treatment alone compared with normal control rats, implying increased oxidative damage to the liver (Figure 4). In contrast, SOD, CAT, and GPx levels were significantly elevated by administration of hispolon to CCl_4_-intoxicated rats, suggesting that it has the ability to restore these enzymes activities in CCl_4_-damaged liver. 

The toxic metabolite CCl_3_ radical binds covalently to the macromolecules and causes peroxidative degradation of cellular membrane leading to the necrosis of hepatocytes [24]. Increase in TBARS levels, evident in our study, suggests enhanced lipid peroxidation leading to tissue damage and failure of antioxidant defense mechanisms to prevent formation of excessive free radicals [25]. Treatment with silymarin and hispolon significantly reversed these changes (Figure 5(a)). 

GSH, a nonenzymatic antioxidant, reduces H_2_O_2_, hydroperoxides (ROOH) and xenobiotic toxicity [26]. GSH is readily oxidized to glutathione disulfide (GSSG) by the reaction with ROOH or xenobiotic compounds that may subsequently cause the reduction of GSH level. The GSSG is either rapidly reduced by GSH reductase and NADPH or utilized in the protein-folding process in the endoplasmic reticulum. There, GSSG is recycled by protein disulfide isomerase to form GSH. Due to these recycling mechanisms, GSH is a particularly efficient intracellular buffer for oxidative stress [27]. Our results indicated that administration of hispolon caused a significant increase in activity of GSH (Figure 5(b)) present in the liver. This may be due to the inhibitory effects on cytochrome P450 and/or promotion of its glucuronidation [28]. GSH constitutes the first line of defense against free radicals and is a critical determinant of the tissue susceptibility to oxidative damage. It has been reported that GSH plays a key role in detoxifying the reactive toxic metabolites of CCl_4_ and that liver necrosis begins when the GSH stores are depleted [29]. In this study, CCl_4_ treatment decreases the hepatic GSH levels and intraperitoneal hispolon pre-treatments restored the hepatic GSH levels to its respective normal value (Figure 5(b)). The effect could be due either to the *de novo* synthesis of GSH, its regeneration, or both.

The liver is a major inflammatory organ, and inflammatory processes contribute to a number of pathological events after exposure to various hepatotoxins. Kupffer cells release proinflammatory mediators either in response to necrosis or as a direct action by the activated hepatotoxins, which are believed to aggravate CCl_4_-induced hepatic injury [7]. TNF-*α*, a pleiotropic proinflammatory cytokine, is rapidly produced by macrophages in response to tissue damage. While low levels of TNF-*α* may play a role in cell protection, excessive amounts cause cell impairment. An increase in the TNF-*α* level has been directly correlated with the histological evidence of hepatic necrosis and the increase in the serum aminotransferase levels [29]. Decicco et al. [30] have reported the stimulation of TNF-*α* production in both serum and liver following CCl_4_ administration, and it is suggested that CCl_3_^*·*^ activates Kupffer cells to release TNF-*α*. TNF-*α* also stimulates the release of cytokines from macrophages and induces the phagocyte oxidative metabolism and NO production. NO is a highly reactive oxidant that is produced through the action of iNOS, and plays a role in a number of physiological processes, such as vasodilation, neurotransmission, and nonspecific host defense [31]. NO can also exacerbate oxidative stress by reacting with reactive oxygen species, particularly with the superoxide anion and forming peroxynitrite. This study confirmed a significant increase in the serum TNF-*α* protein expression after CCl_4_ administration. These alterations were attenuated by the intraperitoneal hispolon pretreatment (Figure 6), which suggests that hispolon suppresses the TNF-*α* protein secretion and/or enhances its degradation.

Overproduction of NO in the liver has been implicated as an important event in endotoxin shock and in other models of hepatic inflammation and injury. NO is known to react with superoxide radical, forming peroxynitrite, an even more potent oxidizing agent [32]. Therefore, this endotoxin shock may alter the balance existing between NO production and its target proteins and enzymes, leading to GSH depletion, free radical generation, and upregulation of iNOS [33]. Previous report demonstrated that CCl_4_ administration increased NO level in the blood plasma or CCl_4_-treated animals [34]. Similarly, under acute CCl_4_ intoxication we revealed considerable NO level in blood plasma (Figure 6(b)) and increased expression of iNOS (Figure 7(a)). Hispolon prevented the accumulation of the plasma NO level in CCl_4_-treated rats. And the treatment with hispolon leads to a reduction in the expression level of both iNOS and COX-2. This may in part be the result of the regulatory activity and expression of NF-*κ*B and need to be proved. 

Monocytes, which are recruited to inflammatory sites, must first extravasate the vasculature by crossing the endothelial basement membrane and transversing the ECM. They migrate toward gradients of signals, which are provided by bacterial products, cytokines, chemokines, ECM proteins, and proteins deposited in the ECM and orient themselves according to the summated effect of these signals [35]. To enable their movement across the ECM, monocytes degrade their proteins by secreting proteases, including MMPs. Among the different MMPs, MMP-9, which is induced strongly by proinflammatory cytokines (e.g., TNF-*α*), is associated specifically with monocyte/macrophage migration [36]. In this study, the expression of protein for MMP-9 in rat liver was induced by CCl_4_. There is evidence for the involvement of MMPs in animal hepatitis models [15]. Others have suggested that MMP-9 participates in the paracetamol-induced hepatotoxicity mediated by sinusoidal endothelial cell injury, which results in the impairment of microcirculation [37]. It is known that MMPs cleave the ECM, leading to the disintegration of tissue integrity and the infiltration of neutrophils and macrophages [38]. The level of protein for MMP-9 in liver, as well as the serum levels of AST and ALT, began to increase 24 h after CCl_4_ treatment, suggesting that MMP-9 was involved in the development of CCl_4_-induced liver damage. In this study, hispolon inhibited the expression of MMP-9 in CCl4-induced liver damage. Moreover, hispolon reduced inflammatory cell staining for MMP-9 in liver damages. This study also suggested that an inhibitory effect of hispolon on liver damage may have been due to the prevention of MMP-9 production in the development of acute hepatitis.

**Figure 9:**
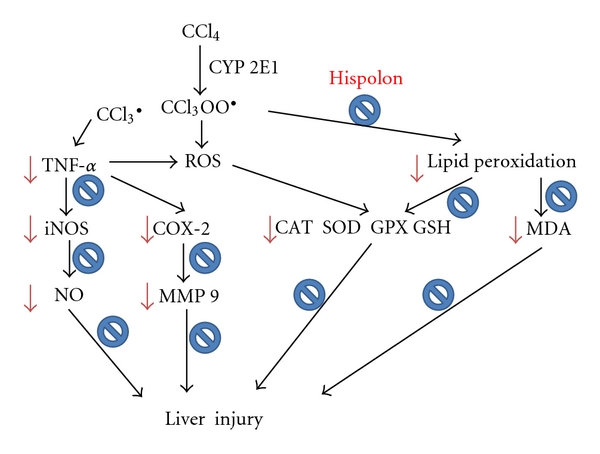
Propose the mechanism of hispolon in CCl_4_-injected rats.

In conclusion, the results of this study demonstrate that hispolon was effective in prevention of CCl_4_-induced hepatic damage in rats. Our results show that the hepatoprotective effects of hispolon may be due to both the inhibition of lipid peroxidation processes and the increase of antioxidant enzymes activity. Hispolon could be also involved in the modulation of proinflammatory cytokines and down-regulation of the expression of MMP-9 (Figure 9). The inhibitory effects of a dietary hispolon may be useful as a hepatoprotective agent against chemical-induced hepatotoxicity *in vivo*.
